# The absence of interleukin 10 affects the morphology, differentiation, granule content and the production of cryptidin-4 in Paneth cells in mice

**DOI:** 10.1371/journal.pone.0221618

**Published:** 2019-09-11

**Authors:** Loni Berkowitz, Catalina Pardo-Roa, Gigliola Ramírez, Omar P. Vallejos, Valentina P. Sebastián, Claudia A. Riedel, Manuel Álvarez-Lobos, Susan M. Bueno

**Affiliations:** 1 Departamento de Gastroenterología, Escuela de Medicina, Pontificia Universidad Católica de Chile, Santiago, Chile; 2 Millennium Institute on Immunology and Immunotherapy, Departamento de Genética Molecular y Microbiología, Facultad de Ciencias Biológicas, Pontificia Universidad Católica de Chile, Santiago, Chile; 3 Millennium Institute on Immunology and Immunotherapy, Facultad de Ciencias de la Vida, Departamento de Ciencias Biológicas, Universidad Andrés Bello, Santiago, Chile; "INSERM", FRANCE

## Abstract

Paneth cells (PCs) are specialized epithelial cells of the small bowel that contain multiple secretory granules filled with antimicrobial peptides and trophic factors, which are essential for the control of the microorganisms growth and maintaining intestinal integrity. Alterations in their function are associated with an imbalance of the normal microbiota, gastrointestinal infections and inflammatory processes, such as Crohn’s disease (CD). One of the most common murine models for studying CD is IL-10^-/-^ mouse. IL-10^-/-^ mice when housed in conventional conditions and take contact with commensal microorganisms develop an acute enterocolitis mediated by a Th1 immune response. Even though, alterations in PCs function are related to CD, they had not been characterized yet in this mouse model. Here we show that in specific pathogen free conditions IL-10^-/-^ mice have aberrant granules and a large number of immature PCs at the bottom of the crypt in the ileum of IL-10^-/-^ mice before developing intestinal inflammation, along with a reduced expression of Indian Hedgehog. In addition, IL-10^-/-^ Paneth cells presented a reduced expression of cryptidin-4, and a heterogeneous distribution of lysozyme+ granules. The alterations in the maturation of the PCs at the bottom of the crypt were not modified after the colonization by the conventional microbiota. On the other hand, depletion of microbiota altered the phenotype, but did not normalize PCs. Our results suggest that IL-10 could be necessary for the integrity of PCs. Moreover, our results help to explain why IL-10^-/-^ mice develop enterocolitis in response to microorganisms.

## Introduction

Crohn’s disease (CD) is an inflammatory bowel disease (IBD) described as a chronic relapsing immunological disorder [1]. Although the pathogenesis of this disease is not completely understood, scientific and clinical evidences suggest that it would be the result of a complex interaction between genetic alterations, innate immune response, imbalance of intestinal microbiota and environmental factors [2]. Specifically, it has been postulated that after a certain stimulus, genetically-susceptible individuals develop an inadequate mucosal immune response against their intestinal microbiota. This response can lead to a pathological inflammation of the digestive tract, mainly involving the terminal ileum and the colon [3].

Some genetic variations have been related with the development of CD, such as mutations on *ATG16L*, *NOD2/CARD15*, *IL23R* and *IL-10* genes [4,5]. It could be related with the role that plays the immune system in the balance between the tolerance to intestinal microbiota and the pathogen control [6,7]. On IBD, the abnormal immune response against microbiota results in a dysfunction of the epithelial barrier and enhanced mucosal permeability [8].

Several *in-vivo* models have been developed to study the pathogenesis and the immune response of IBD [9]. One of this is the interleukin-10 deficient mice (IL-10^-/-^). IL-10^-/-^ mice is able to develop a slow and progressive enterocolitis under specific pathogen-free (SPF) conditions [10]. Supporting the importance of the interactions between the microbiota and immune system on IBD is the observation that IL-10^-/-^ mice maintained under germ-free (GF) conditions neither develop histologic evidence of disease nor immune activation [11]. However, when these mice are stimulated with commensal bacteria, progressively develop more aggressive colitis after one to four weeks of colonization [11,12].

IL-10 may be considered the most important anti-inflammatory cytokine in humans and its main role is to reduce the secretion of pro-inflammatory cytokines to protect the tissues against excessive immune responses and cellular damage [5].

Recently, mutations in IL-10 or components of its signaling pathway have been involved in the pathogenesis of IBD. For example, mutations in the leader sequence of IL-10 protein and IL-10-1082A/G polymorphism are related with the severity of CD [13][14]. Moreover, the presence of IL-10 polymorphisms as rs304496 and *IL-10* epistatic interaction with genes of STAT3 signaling pathway, has been related with the risk of pediatric IBD [15].

Among the factors involved in the maintenance of intestinal homeostasis, the Paneth cells (PC) stand out. These specialized epithelial cells of the small bowel produce and secrete antimicrobial peptides and trophic factors, in order to control the excessive microorganism growth and to aid intestinal repair [16]. Imbalances of the composition of the microbiota, gastrointestinal infections and inflammatory processes have been associated to alterations in the function of PCs [17]. Moreover, functional alterations of PCs have been postulated as a possible trigger for CD [18]. Interestingly, these cells have not yet been characterized in IL-10^-/-^ mice, so their contribution to the enterocolitis developed by these mice is still unknown. Considering that enterocolitis does not occur if IL-10^-/-^ mice are in germ-free conditions [10], it is possible that inflammation depends on an altered interaction between the microbiota and the immune response. Given the aforementioned contentions, the aim of this study was to evaluate and characterize PCs in the ileum of IL-10^-/-^ mice. Here we observed that IL-10^-/-^ mice have histological and functional abnormalities in PCs. These results suggest that alterations in PCs may contribute to the enterocolitis developed by IL-10^-/-^ mice and that IL-10 could be necessary for the integrity of PCs. Moreover, our results suggest that the alterations observed in the PC could be due to a deregulation of the process of proliferation and differentiation of the secretory cells in the crypt, even before the development of inflammation.

## Materials and methods

### Ethics statement

All the experiments using mice were conducted in agreement with the ethical standards and according to the local animal protection law. All experimental protocols were reviewed and approved by the Scientific Ethical Committee for Animal and Environment Care of the Pontificia Universidad Católica de Chile (Protocol 170329009).

### Mice strains

Seven-week-old C57BL/6 wild-type male mice (WT) and B6.129P2.Il10^tm1Cgn^/J mice (IL-10^−/−^) were originally purchased from Jackson Laboratories (Bar Harbor, ME, USA) and maintained in the specific pathogen-free (SPF) animal facility at the Facultad de Ciencias Biológicas, Pontificia Universidad Católica de Chile. Specifically, mice were housed separated by genotype in cages individually ventilated with HEPA filter and living under 12 light/12 dark cycle condition. Mice were given ad libitum access to sterile food (LabDiet^®^) and autoclaved water.

### Tissue treatment for histological procedures

Mice were euthanized with isoflurane anesthesia (5%) and then they were cervical dislocated. Distal ileum was dissected and perfused with 2 mL sterile phosphate-buffered saline (PBS, pH 7.4), and then fixed in 10% formalin solution. Tissues were embedded in paraffin (Tissue Processor Leica ASP300), and transversal sections of 5 mm were adhered to positively charged glass slides, deparaffinized, and used for hematoxylin-eosin (H&E), alcian blue-PAS (AB-PAS), and immunofluorescent (IF) staining.

### Histopathology and morphometrics analysis

For histopathology analyses, sections were stained with H&E or AB-PAS (pH 2.5) by routine methods. The histological score was evaluated in a blinded fashion on H&E-stained transversal sections of the terminal ileum, resulting in a score from 0 (non-inflamed) to 12 (highly inflamed) per section as previously described [19]. The number of PC per crypt was examined in ileum sections stained with AB-PAS using a magnification of 100X under immersion oil. This staining, allows to visualize the granules of PC in magenta (PAS positive), while the mucus of Goblet cells stain blue (Alcian-Blue positive) [20]. At least 20 crypts were assessed per section by a single blinded observer, analyzing 3–5 sections per mouse. Also, the number of intermediate cells (AB+ and PAS+) was assessed and classified according to their location. Thus, adjacent to PCs (P1), in the middle zone of proliferation (P2), or in upper positions over differentiated Goblet Cells (P3) (described in S1 Fig).

### Immunofluorescent staining

After deparaffinization, antigen demasking was performed by boiling the samples in 0.01M Sodium Citrate Buffer (pH 6.0)–Tween 0.05% for 45 min. After cooling them down at RT, slides were washed 2 times with distilled H_2_O for 5 min each, and then 3 times in TBS-Tween 0.1% for 5 min each. Sections were blocked with 50ml of TBS-Tween 0.1%—FBS 5%—BSA 10%, for 60 min at RT in a humidified chamber. Then, the slides were incubated overnight at 4°C with primary antibody against Lysozyme (goat anti- Lysozyme C 1:200, Santa Cruz catalog no. sc-27958). Fluorochrome-conjugated secondary antibody, Donkey anti-goat IgG (H+L) Cross-Adsorbed Alexa Fluor 488 (Invitrogen, catalog no. A11055, diluted 1:200), was used to incubate the slides for 1 h at RT in a humidified chamber. Sections were mounted using Vectashield Antifade Mounting Medium with DAPI (Vector Laboratories, catalog no. H-1200), and visualized using an epifluorescence microscope (Nikon Eclipse E200).

### Transmission electron microscopy analyses

Sections of the distal ileum (1 mm) were fixed for 16 h by immersion in 2.5% glutaraldehyde in 0.1 M cacodylate buffer (pH 7), and then washed three times with cacodylate buffer. The sections were post-fixed with 1% osmium tetroxide (OsO_4_) for 90 min, and washed 3 times with bidistilled water. Then, the sections were treated with 1% aqueous uranyl for 1 h, and sequentially dehydrated through graded acetones. Sections were left overnight in epon/acetone 1:1 and then in pure resin for 4 h. Finally, sections were included in fresh resin and polymerized in an oven at 60 ° C for 24 h. Ultrafine sections (80 nm) were obtained using an ultramicrotome Leica Ultracut R, which were incubated with 4% uranyl acetate in methanol for 1 minute and lead citrate for 5 minutes. The grids were examined using a Phillips Tecnai 12 electron microscope, operated at 80 kV and at a magnification of 1,700–2,250X. This work was performed by the Advanced Microscopy Facility at Pontificia Universidad Católica de Chile.

### Real time PCR

The mRNA levels of *cryptdin-1*, *cryptdin-4*, *reg3γ*, *lysozyme*, *tnfα* (Life technologies, catalog no. Mm00443260_g1), *ifnγ* (Life technologies, catalog no. Mm01168134_m1), *ihh* (Life technologies, catalog no. Mm01259021_m1) and *wnt5a* (Life technologies, catalog no. Mm00437347_m1) were measured by quantitative real-time PCR analysis. Briefly, total RNA was extracted from distal ileum (4cm) using TRIZOL^®^ reagent (Invitrogen, catalog no. 15596026) according to the manufacturer’s instructions, and then treated with DNase I Amplification Grade (Invitrogen, catalog no. 18068015) for eliminating DNA. For the quantification of the mRNA levels of antimicrobial peptides, a total of 1μg RNA was reverse transcribed to cDNA by using the iScript RT Supermix (Biorad, catalog no. 1708890). The resulting cDNA was amplified by real-time PCR with a StepOnePlus thermocycler (Applied Biosystems, CA) using the SsoAdvanced Universal SYBR Green Supermix (Biorad, catalog no. 1725270). The primers are listed in S1 Table in Supporting information. The amplification conditions were as follows: 30 sec at 95 °C and 40 cycles of 15 sec at 98 °C and 1 minute of annealing and extension at 56°C. On the other hand, the mRNA levels of *tnf-α*, *ifn-γ*, *ihh* and *wnt5a* were quantified using TaqMan^®^ RNA-to-Ct^™^ 1-Step Kit (Life technologies, catalog no. 4392938), according to the manufacturer’s recommendations. The amplification conditions were as follows: 1 cycle of 15 min at 48°C for reverse transcription, 1 cycle of 10 min at 95°C for enzyme activation, 40 cycles of 15 sec at 98 °C followed by 1 minute at 56°C for denaturation, annealing and extension.

The relative quantification values were calculated using a comparative threshold cycle (2^−ΔΔct^) program on StepOne software. For this, *gapdh* or *β2m* (Life technologies, catalog no. Mm00437762_m1) were used as housekeeping genes, and the average value obtained by the wild-type mice was used as control.

### Clinical signs of colitis in response to conventional microbiota

IL-10^-/-^ mice were maintained under SPF conditions or in the presence of conventional microbiota for 2 weeks. Every two days, they were evaluated and classified based on the apparition of perianal edema, rectal inflammation, diarrhea, mucus in stools and rectal prolapse according their clinical score of colitis signs (described in S2 Table in Supporting information).

### Depletion of gut microbiota

Intestinal microbiota of 9-week-old IL-10^-/-^ mice was depleted by administering broad-spectrum antibiotics (ATB group) via autoclaved drinking water for two weeks. Broad-spectrum antibiotics included 0.5 g/L vancomycin, 1 g/L ampicillin, 1 g/L kanamycin and 1 g/L metronidazole supplemented with 1% (wt/vol) glucose. The drinking water for control group was prepared in the same way only without antibiotics (VEH group). The drinking water was changed three times per week. The antibiotic treatment did not affect body weight or water intake. Depletion of gut microbiota was verified by evaluating bacterial load in stool samples.

### Statistics analysis

Statistical analyses were performed using Prism v6 (GraphPad Software, San Diego, CA, USA). Unpaired Student’s *t*-test was used to assess whether the means of two normally distributed groups differed significantly, and Mann Whitney U-test as its non-parametric counterpart. One-way ANOVA with Tukey post-test was used to compare multiple means. Two-way ANOVA analysis with repeated measures was also used in some experiments, with Bonferroni’s multiple comparisons post-test.

## Results

### Interleukin-10 deficient mice present abnormal PCs

**Fig 1:**
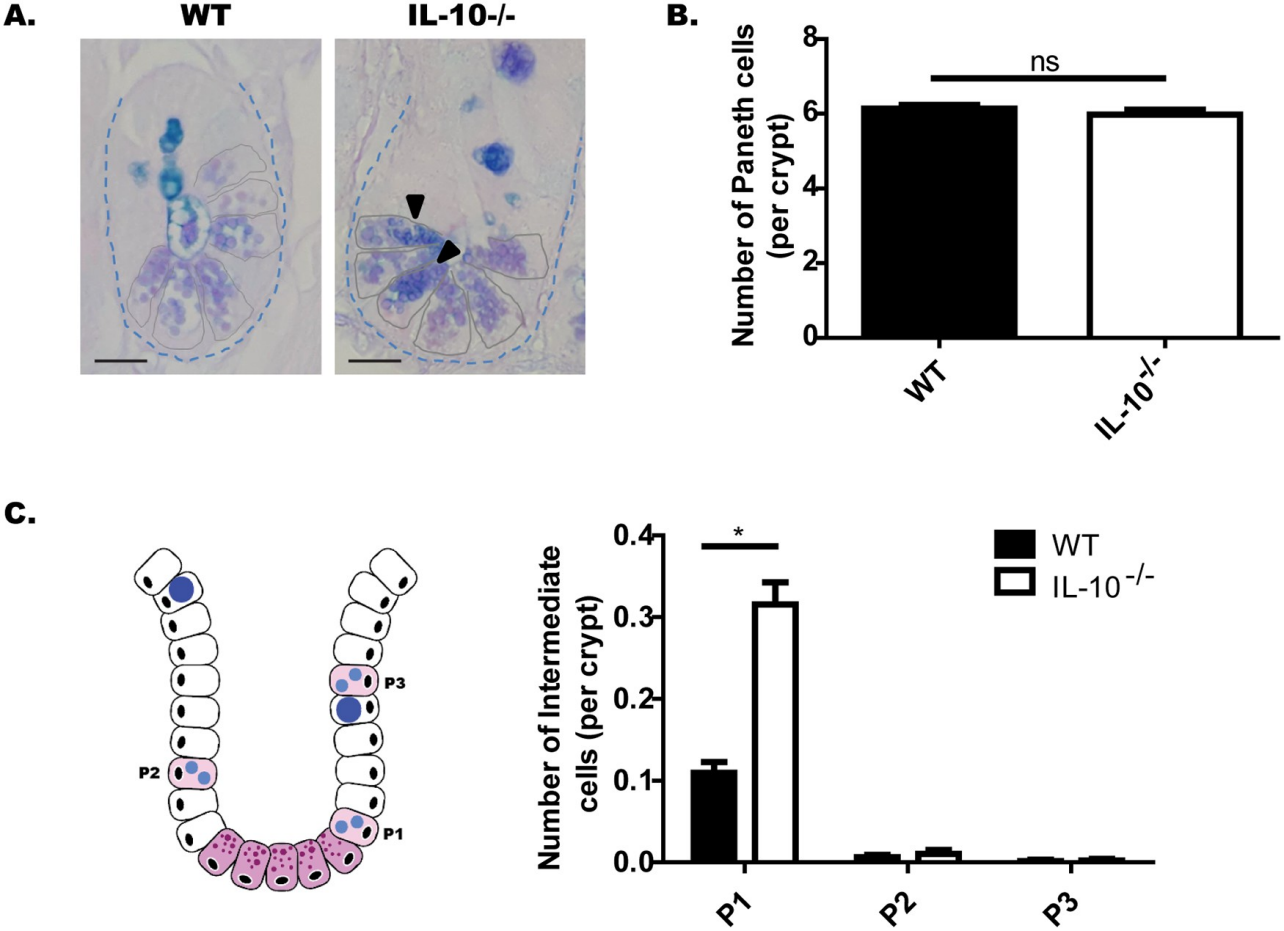
IL-10^-/-^ mice have more immature Paneth cells at the bottom of the crypt. (A) Representative images of Lieberkühn crypts stained with AB-PAS, of C57BL/6 and IL-10^-/-^ mice. The black arrows show Paneth cells with intermediate cell staining AB+PAS+. Scale bars are 25μm. (B) Number of PC per crypt. n = 120 crypts were analyzed from 4 mice. (C) Quantification of Intermediate Cells per crypt of both strains, according to their location: adjacent to Paneth cells (P1), in the middle zone of proliferation (P2), or in upper positions over differentiated Goblet Cells (P3) (2-way ANOVA, post-hoc Bonferroni *p˂0.05, ***p ˂0.001, n = 660 crypts).

In order to evaluate the morphology and number of PCs, ileum sections of WT and IL-10^-/-^ mice were stained with alcian-blue PAS (AB-PAS). Using this technique, as described in materials and methods, secretory granules of PCs show a magenta staining (PAS+), while mucin of Goblet cells shows a blue staining (AB+) [20]. According to the crypts of Lieberkühn analyses, no significant differences were observed in terms of morphology (Fig 1A), nor in the number of PCs per crypt (Fig 1B) between both strains. Interestingly, numerous PCs in IL-10^-/-^ mice present granules that appear more compact and stain positive for both AB and PAS. These PCs, which share characteristics with Goblet cells are designated as intermediate cells, and these cells are considered precursor forms [21]. They originate from the stem cells located in the crypt and remain in the proliferation zone until they differentiate into PCs, when they settle at the bottom of the crypt [21,22]. Considering this, we decided to classify them according to their location: adjacent to PCs (P1), in the middle zone of proliferation (P2), or in upper positions over differentiated Goblet Cells (P3) (The characterization is described in S1 Fig). According to these results, IL-10^-/-^ mice showed significantly more intermediate cells than WT mice, particularly at the bottom of the crypt (Fig 1C and 1D).

**Fig 2:**
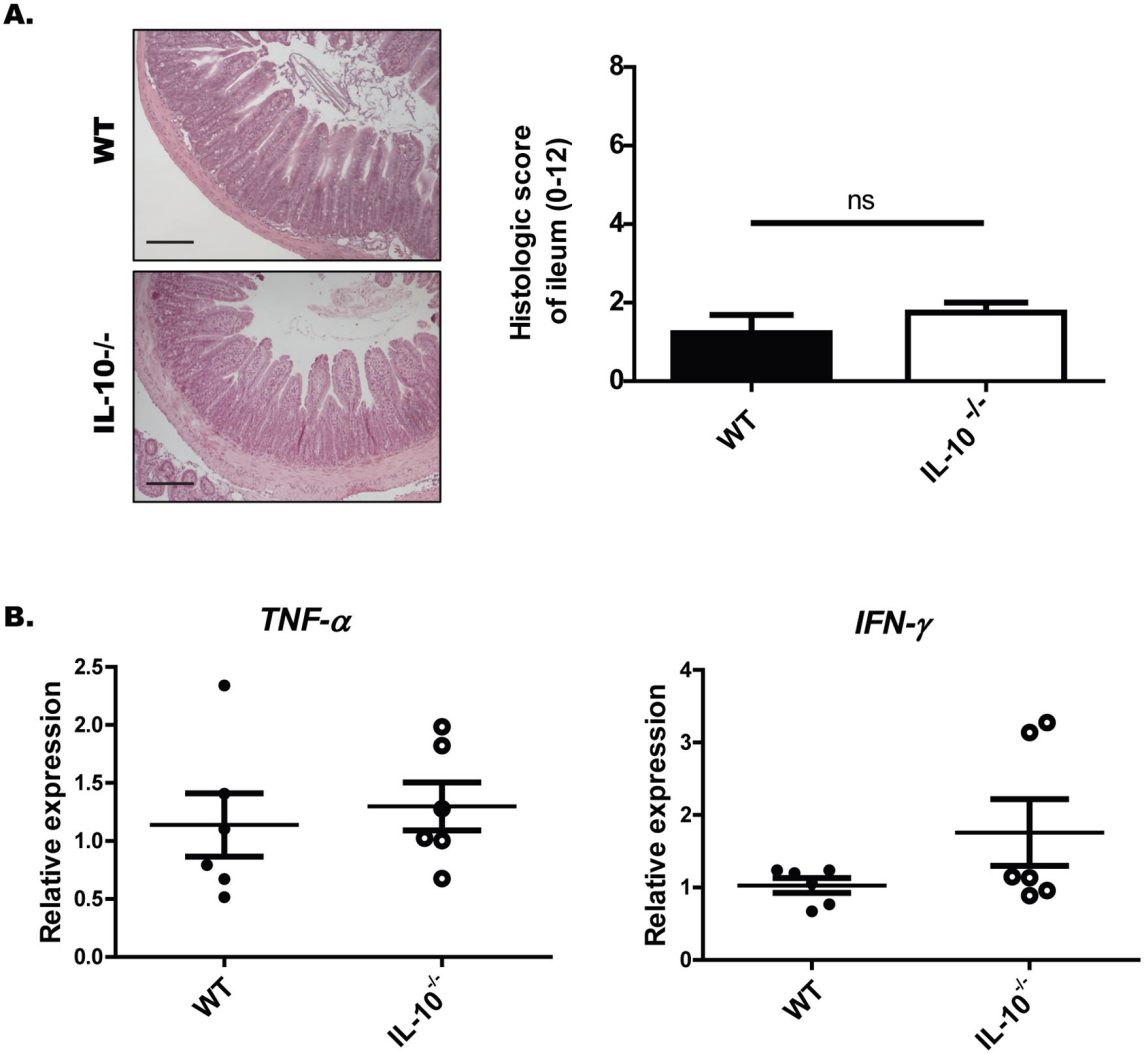
Seven-week-old IL-10^-/-^ mice do not present more signs of ileum inflammation than WT mice under SPF conditions. (A) Histopathological analysis of ileum sections of wild-type and IL-10^-/-^ mice. Left panel: Representative images of ileum sections stained with H&E (Scale bars are 200μm). Right panel: Histologic score. (B) Relative expression of *TNF-α* and *IFN-γ* in ileum sections of WT (n = 5) and IL-10^-/-^ (n = 6) mice.

In order to evaluate whether these alterations could be explained by tissue inflammation, histopathological analyzes of HyE-stained sections of the ileum were performed. From these analyses it was possible to observe that at the time of the study IL-10^-/-^ mice did not present enterocolitis or more signs of ileum inflammation than WT mice (Fig 2A). In fact, IL-10^-/-^ mice have similar levels of IFN-γ or TNF-α compared to WT mice. Only two of six IL-10^-/-^ mice showed high levels of IFN- γ mRNA (Fig 2B), suggesting that these mice were beginning to develop ileal inflammation.

### The secretory granules in PCs from IL-10-/- mice have an aberrant ultrastructure

**Fig 3:**
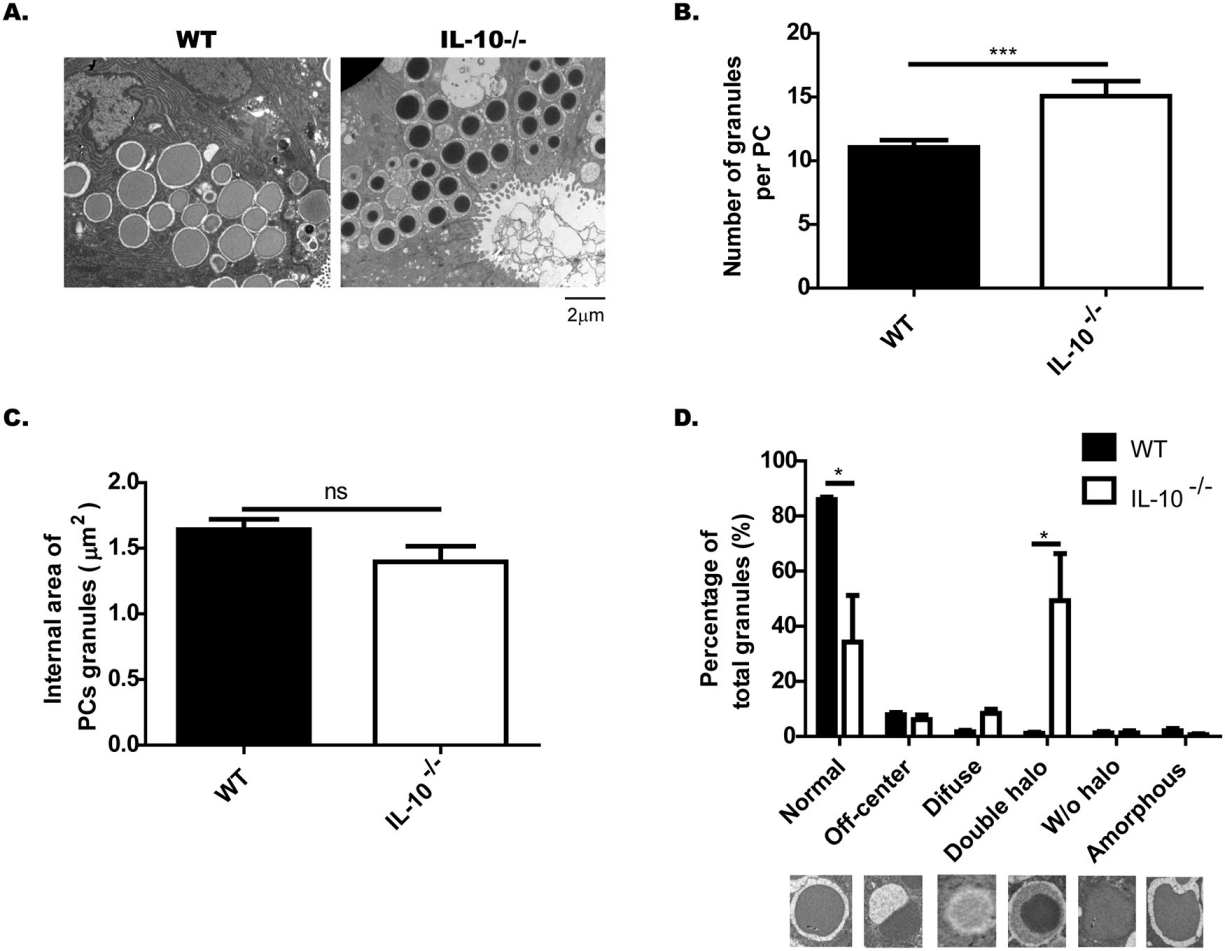
IL-10^-/-^ mice present abnormal Paneth cells granules. Paneth cell (PCs) granules of C57BL/6 and IL-10^-/-^ mice were evaluated by transmission electron microscopy (n = 3). (A) Representative images of PC granules from both strains (4200X, Scale is 2μm). (B) Number of granules per PC (t-student, ***˂p 0.001). (C) Area of the electrondense core of the PC granules. D) Classification of granules, according to their TEM morphology in: normal, off-center, diffuse, double halo, without halo and amorphous (n = 3 mice, 2-way ANOVA, post-hoc Bonferroni *p˂0.05).

To evaluate in more detail the secretory granules in PCs of IL-10^-/-^ mice, sections of distal ileum of both strains were analyzed by transmission electron microscopy. Interestingly, it was possible to observe an abundant number of abnormal granules in the PCs of IL-10^-/-^ mice (Fig 3A). Using morphometric analysis, it was possible to conclude that IL-10^-/-^ mice have a higher number of granules per PCs than WT mice (Fig 3B). Although the electron-dense core of these granules seems to be smaller than the electron-dense core of the WT granules, these differences were not statistically significant (Fig 3C).

On the other hand, after classifying the granules according to their morphology, it was possible to verify that IL-10^-/-^ mice presented a significant lower number of normal granules than WT mice. This can be explained to a large extent by the high percentage of granules with double halo (Fig 3D). These granules are characterized by having a core of higher electron-density than normal granules and a wide halo of semi-dense content surrounding this core. Moreover, these granules are very similar to those described in intermediate cells [23].

In order to evaluate whether this phenotype is the result of an alteration of the epithelium development or the allocation of the secretory lineages, the intestinal stem cells (ISC) were characterized in IL-10^-/-^ mice.

**Fig 4:**
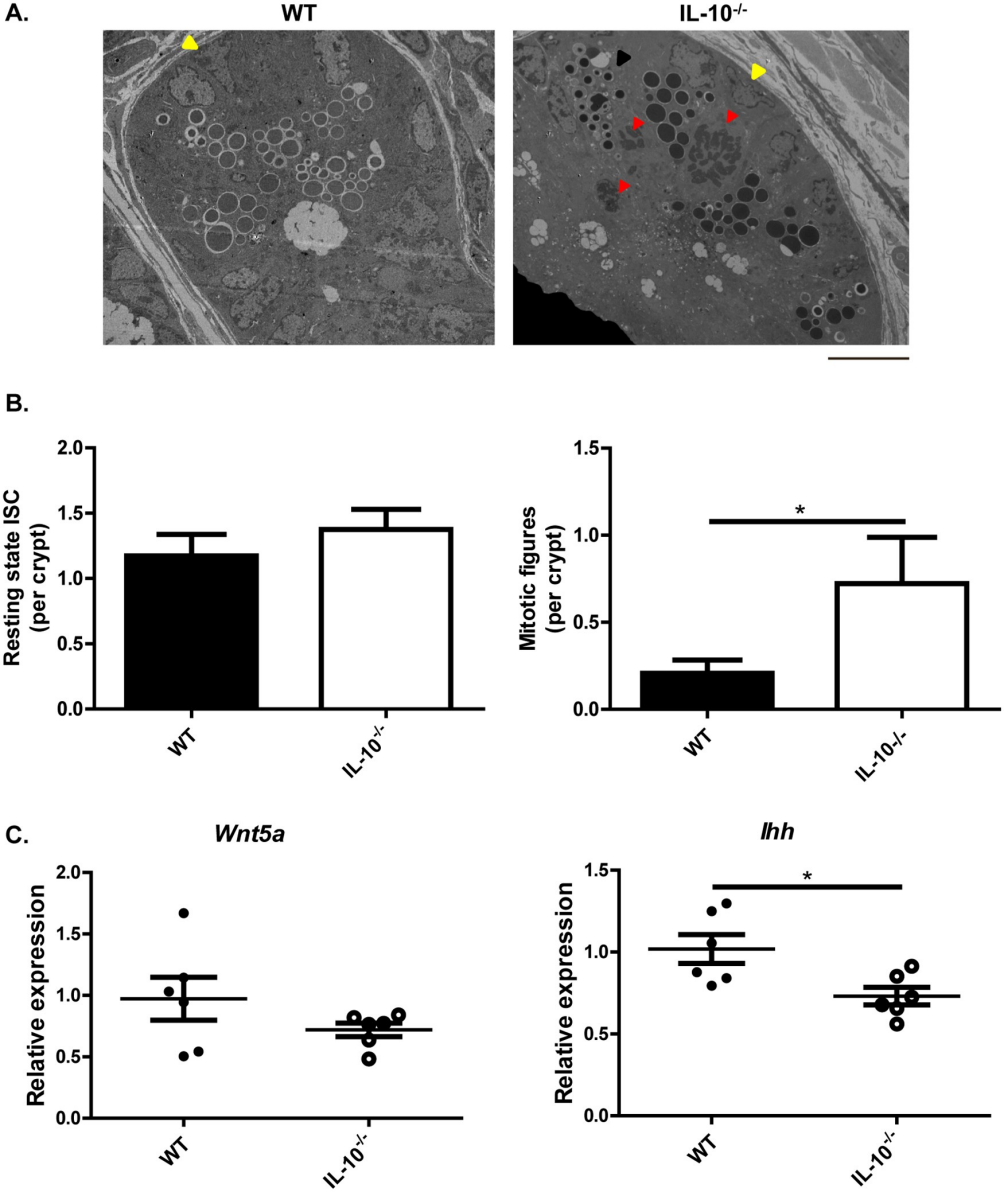
The crypts of IL-10^-/-^ mice have increased cell proliferation and alterations in secretory lineages allocation. **(A)** Ileum crypts of C57BL/6 and IL-10^-/-^ mice evaluated by transmission electron microscopy (scale bar is 10μm). In both strains were observed basal intestinal stem-cells (ISC) in resting state (yellow arrows) and undifferentiated cells in mitotic phase (red arrows). The black arrow shows a Paneth cell with intermediate granules located in the bottom of the crypt of an IL-10^-/-^ mouse. (B) Quantitative analysis of the number of basal ISC in resting state and undifferentiated cells in mitotic phase (red arrows) present in the crypts of C57BL/6 (n = 29 crypts) and IL-10^-/-^ (n = 20 crypts) mice (t-test *p˂0.05). (C) Relative expression of *Wnt5a* and *Ihh* in ileum sections of WT (n = 5) and IL-10^-/-^ (n = 6) mice (U-test Mann Whitney *p˂0.05).

The same sections analyzed by electronic microscopy in Fig 3 were also analyzed for the morphology and the number of ISC present in the crypts of wild-type and IL-10^-/-^ mice (Fig 4). In both strains, basal ISC in resting state (yellow arrows) and undifferentiated cells in mitotic phase (red arrows) were observed (Fig 4A). According to the quantitative analyzes, similar number of resting basal ISC were observed between the two mice strains (Fig 4B, left). However, IL-10^-/-^ mice had a significantly higher number of undifferentiated cells in mitotic phase compared to wild-type mice (Fig 4B, right). The mRNA expression levels of Wnt5a and Indian Hedghog (Ihh) were evaluated in IL-10^-/-^ mice given these transcription factors are relevant for the proliferation of stem cells and differentiation of PCs. According to the results, IL-10^-/-^ mice had similar mRNA levels of Wnt5a compared to WT mice (Fig 4C, left). However, IL-10^-/-^ mice showed significantly lower levels of Ihh mRNA (Fig 4C, right) compared to wild-type mice. This transcription factor is mainly expressed by mature PC at the crypt level and limits the proliferation of PCs precursors [24]. Therefore, the lower expression of Ihh detected in IL-10^-/-^ mice is consistent with the high number of immature PC present in this strain.

### Interleukin-10 deficient mice exhibit an altered expression of bactericidal peptides

**Fig 5:**
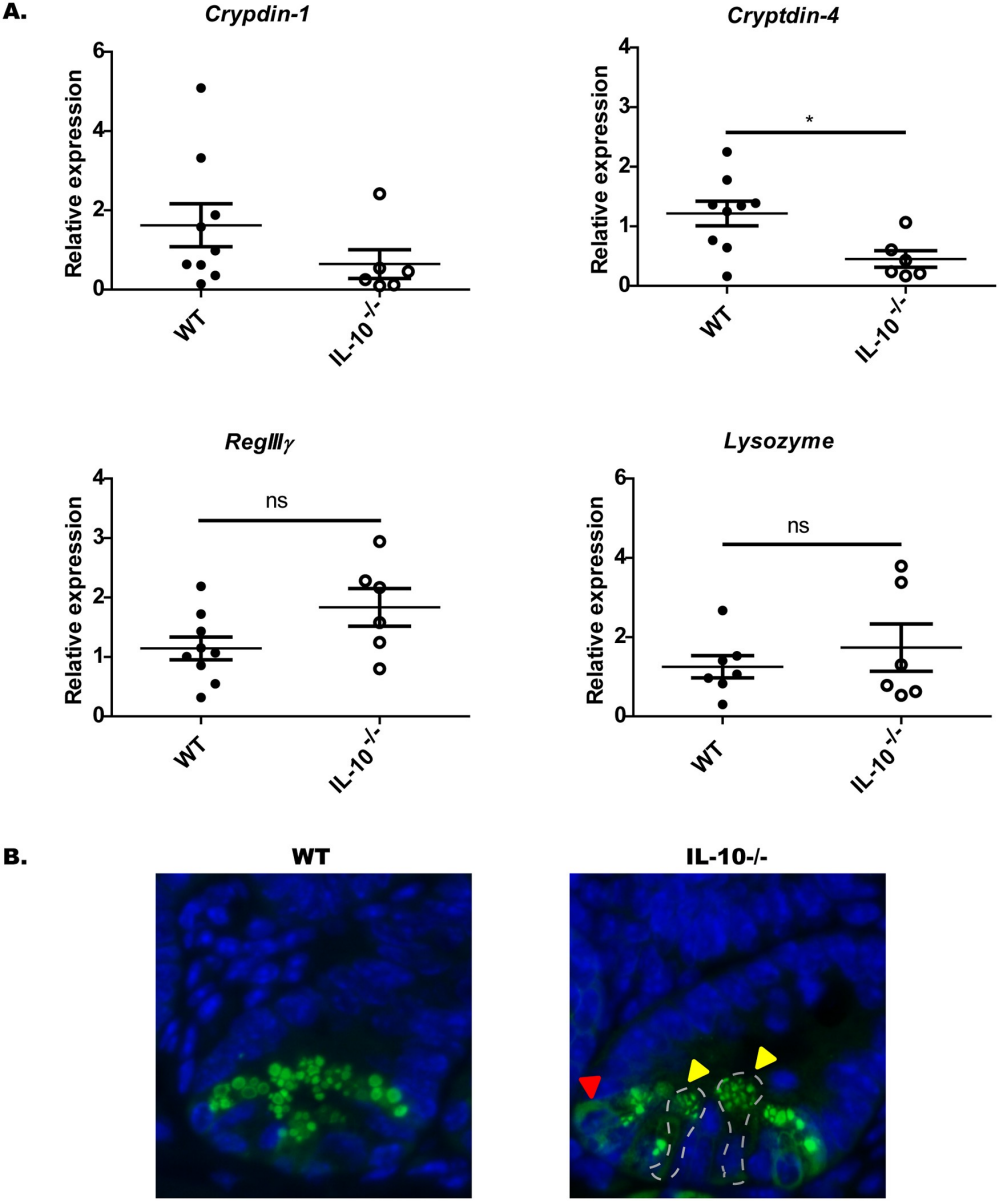
Paneth cells of IL-10^-/-^ mice had a reduced expression of Cryptdin-4 mRNA transcripts and an abnormal packing of Lysozyme. (A) Relative expression of *cryptdin-1*, *cryptdin-4*, *RegIIIγ* and *lysozyme* mRNA in ileum sections of C57BL/6 (n = 9) and IL-10^-/-^ (n = 6) mice (t-student, *p˂0.05). (B) Representative images of lysozyme immunodetection in Lieberkühn crypts of C57BL/6 and IL-10^-/-^ mice. Yellow arrows indicate abnormal granules size and distribution. Red arrow indicates diffuse cytoplasmic content.

To assess the effect of IL-10 deficiency over the content of bactericidal peptides in PCs, bactericidal peptides were analyzed by qRT-PCR in these cells from IL-10^-/-^ and WT (Fig 5). Particularly, we analyzed peptides like cryptdins and lysozyme that are specific peptides of PC, while RegIIIγ is also expressed in the rest of the epithelium [25].

According to the analysis of the results obtained by qRT-PCR, a significant reduced gene expression of cryptidin-4 was detected in IL-10^-/-^ mice as compared to WT mice (Fig 5A). Even though, the levels of cryptydin-1 mRNA tended to be lower in IL-10^-/-^ mice compared to WT mice there was not significant difference between these two groups (Fig 5A). Regarding the gene expression of lysozyme and RegIIIγ, no differences were found between both mice strains (Fig 5A).

On the other hand, in order to analyze the packing of the granular content, the presence of the lysozyme was evaluated by immunofluorescence of ileum sections [20]. According to these results, and consistently to the results obtained previously, IL-10^-/-^ mice did not present differences in the number of cells positives for lysozyme (Lys+). However, the presence of granules of heterogeneous sizes and variable distribution of this peptide was observed between different PCs of IL-10^-/-^ mice. This was not observed in WT mice which, granules were circular and uniform (Fig 5B).

**Fig 6:**
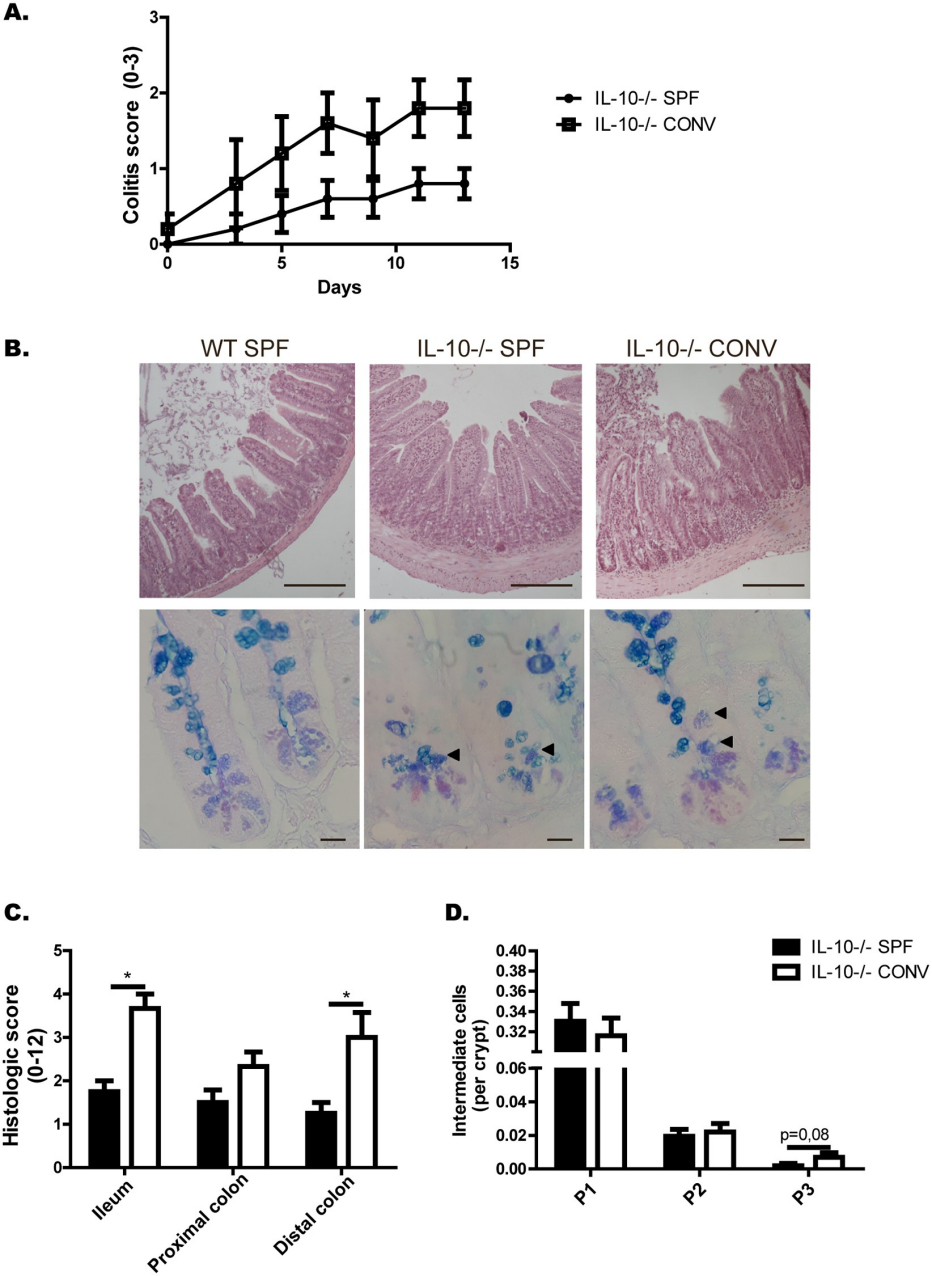
Conventional microbiota induces enterocolitis in IL-10^-/-^ mice without affecting the number of intermediate cells at the bottom of the crypt. (A) Colitis score of IL-10^-/-^ mice under SPF conditions or in the presence of conventional microbiota (n = 5). (B) Representative images of ileum sections stained with H&E (upper panel, scale bars are 200μm) and with AB-PAS (lower panel, scale bars are 25μm) from WT mice or IL-10^-/-^ mice under SPF conditions or in the presence of conventional microbiota. (C) Histologic score of ileum, proximal colon and distal colon of IL-10^-/-^ under SPF conditions or in the presence of conventional microbiota (t-student, *p˂0.05, n = 4). (D) Quantification of Intermediate Cells (AB^+^PAS^+^) per crypt, according to their location: adjacent to Paneth cells (P1), in the middle zone of proliferation (P2), or in upper positions over differentiated Goblet Cells (P3) (n = 1000 crypts).

In order to evaluate the role of the microbiota in the phenotype observed and to rule-out that the results are due to the IL-10^-/-^ mice could harbor a colitogenic microbiota. The number of intermediate cells per crypt in IL-10^-/-^ mice was analyzed after these mice were exposed to specific-pathogen-free conditions (SPF) or to an inflammatory environment as it is conventional microbiota (Fig 6). According to the results, IL-10^-/-^ mice exposed to conventional microbiota presented more severe intestinal inflammation and signs of colitis than IL-10^-/-^ mice maintained under SPF conditions (Fig 6A). Intestinal inflammation and the number of intermediate cells were also analyzed by histology of intestinal sections stained with H&E and AB-PAS, respectively (Fig 6B). Ileum and distal colon showed significant higher score of intestinal inflammation in those mice exposed to conventional microbiota than those IL-10^-/-^ mice maintained under SPF conditions (Fig 6C). Interestingly, mice IL-10^-/-^ exposed to conventional microbiota had similar number of intermediate cells per crypt compared to IL-10^-/-^ mice maintained under SPF (Fig 6D). There is only a slight increase in the number of intermediate cells in higher positions in response to the conventional microbiota. This phenomenon normally occurs in reparative processes of the crypt, where there is an expansion of intermediate cells to replenish the damaged epithelium [21].

**Fig 7:**
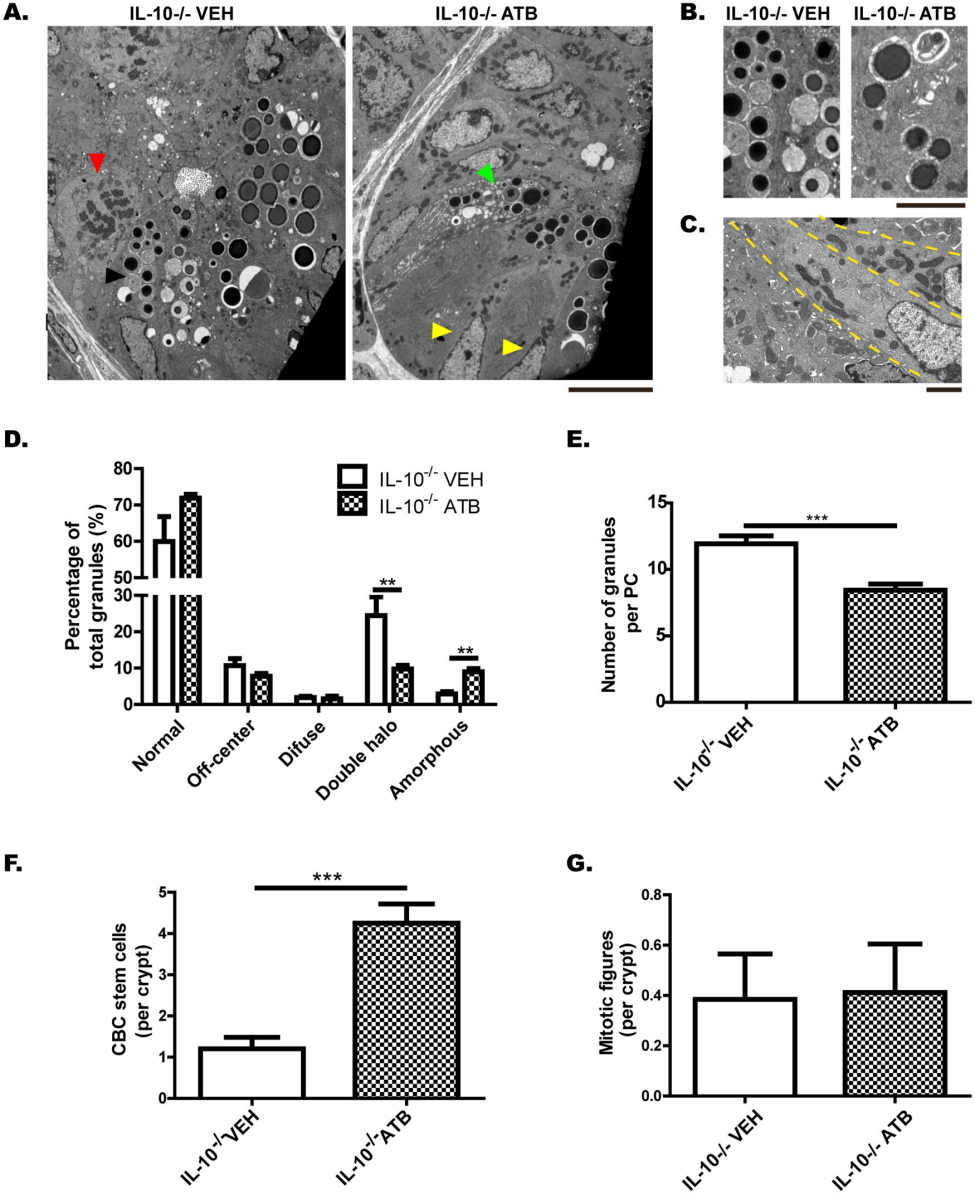
Depletion of gut microbiota partially improves the Paneth cells phenotype, but does not normalize the state of the crypt. **(A)** Ileum crypts of IL-10^-/-^ mice treated with vehicle (IL-10^-/-^ VEH) or broad-spectrum antibiotics (IL-10^-/-^ ATB), evaluated by transmission electron microscopy (scale bar is 10μm). Black arrow shows a PC with double-halo granules, green arrow shows a PC with amorphous granules, yellow arrows show Crypt Base Columnar (CBC) stem cells and red arrow show a mitotic figure. (B) Representative images of PC granules from both groups (scale bar is 5 μm). (C) Crypt Base Columnar (CBC) stem cells of a IL-10^-/-^ ATB mouse, with large amounts of mitochondria in their cytoplasm (scale bar is 2 μm). D) Classification of granules according to their TEM morphology in: normal, off-center, diffuse, double halo and amorphous (n = 3 mice, 2-way ANOVA, post-hoc Bonferroni *p˂0.05). E) Number of granules per PC (t-student, ***˂p 0.001). F) Number of CBC stem cell per crypt (t-student, ***˂p 0.001). G) Number of undifferentiated cells in mitotic phase per crypt.

On the other hand, we characterized PCs and ISCs of microbiota-depleted mice (Fig 7). For this, 9-week-old mice received a broad-spectrum antibiotic treatment (IL-10^-/-^ ATB) or vehicle (IL-10^-/-^ VEH) for two weeks. Electron microscopy images showed that IL-10^-/-^ mice lacking intestinal microbiota did not differ in the number of PCs per crypt (data not shown), but had significantly fewer granules with double halo than IL-10^-/-^ VEH mice (Fig 7A, 7B and 7D). In addition, IL-10^-/-^ ATB mice had significantly fewer granules per PC than IL-10^-/-^ VEH mice (Fig 7A and 7E). Despite this, antibiotic treatment did not normalize the niche of PCs. Although their PCs did not have an immature phenotype, they had significantly more amorphous granules (Fig 7A, 7B and 7D). In addition, the crypts of IL-10^-/-^ mice lacking intestinal microbiota had significantly more Crypt Base Columnar (CBC) stem cells, with large amounts of mitochondria in their cytoplasm (Fig 7A, 7C and 7F). The number of mitotic figures per crypt did not differ between the two groups (Fig 7G).

## Discussion

In this study we have shown that under SPF conditions IL-10^-/-^ mice have alterations in maturation of PCs, even before developing intestinal inflammation, including abnormal granules and a reduced expression of cryptidins. To our knowledge, this is the first study describing these alterations in mice lacking IL-10. The alterations observed in PC could contribute to the enterocolitis observed in this model of intestinal inflammation. Moreover, this study suggests that IL-10 is necessary to maintain the integrity of PC in the presence of microorganisms.

A previously published study reported a reduced number of PCs in old IL-10^-/-^ mice raised under SPF conditions [26]. We did not observe a reduced number of PCs in this strain, compared to the WT [26]. However, we observed a large number of immature PCs at the bottom of the crypt, with aberrant granules and reduced expression of cryptdin-4 and a tendency also for a reduction in cryptdin-1. It is possible that in the long term these aberrations lead to cell death that cannot be compensated.

In addition, IL-10^-/-^ mice in SPF conditions presented a significantly higher number of undifferentiated cells in mitotic phase and lower levels of *Ihh* mRNA than WT mice. This transcription factor is mainly expressed by mature PCs at the crypt level and limits secretory lineage allocation [24]. It has been demonstrated that in the presence of epithelial damage the expression of Ihh decreases and the expression of Wnt ligands increases, in order to favor the development of intermediate secretory cells [21]. Interestingly, we did not observe an increase in the number of PCs in this strain. Therefore, it seems that there is an accelerated renewal of these cells, even before reaching their complete differentiation.

On the other hand, we did not observe an increase in Wnt5a in the IL-10^-/-^ mice, one of the possible ligands involved in the proliferation of secretory cells. However, the homeostasis of the crypt depends on various factors that favor or restrict ISC self-renewal including cytokines, matrix proteins, and growth factors [27]. It is demonstrated that Ihh stimulates the expression of bone morphogenetic proteins (BMPs) in mesenchymal cells, which normally acts to inhibit ISC self-renewal [27]. In fact, high concentration of BMPs inhibits Wnt-β-catenin signaling to ensure a balanced control of ISC self-renewal, allocation and differentiation [28]. Therefore, the reduction of Ihh could favor a reduced expression of BMPs, with a consequent imbalance in the self-renewal of ISC and an increase of intermediate cells.

Moreover, it has been shown that lower expression of Ihh correlates with a greater expression of metalloproteinases [27], which in response to damage will favor tissue remodeling. However, increased expression of these enzymes in the long term could lead to a permanent damage [29].

Taking all of this together, these results suggest that IL-10 is necessary to maintain the homeostasis of the crypt, since its absence alters the ISC self-renewal and PC differentiation. In this context, a previous study showed that the promotion of cell differentiation of secretory lineages in IL-10^-/-^ mice using metformin improved the function of the epithelial barrier [26]. Furthermore, one study demonstrated the importance of IL-10 in the induction of the secretion of WNT1-inducible signaling protein 1, a protein involved in the proliferation and repair of the intestinal epithelium [30]. However, up until now not much is known about the role of IL-10 in the differentiation of the intestinal epithelium.

On the other hand, it is possible that IL-10 signaling is also required to maintain a correct functioning of these PC cells, given that their intense secretory activity is very sensitive to endoplasmic reticule stress or to autophagy defects [18]. In fact, mutant mice in both pathways have aberrant secretory granules and develop an inflammation that resembles the alterations observed in patients with CD [18]. Interestingly, it has been demonstrated that IL-10 is essential to avoid endoplasmic reticule stress in Goblet cells [31], another type of secretory cell present in the intestinal epithelium. Moreover, the granules observed in IL-10^-/-^ mice are very similar to those present in other mutant models, where pathological autophagy processes have been detected, such as Irgm1 deficient mice [23]. In fact, a study showed that IL-10 would be important to avoid the pathological autophagy present in other pathologies, through the activation of the Akt / mTORC1 pathway [32]. Together, these observations suggest that IL-10 is important to avoid pathological processes in PCs and to maintain their normal functioning. This is particularly consistent with the results obtained in the presence and absence of gut microbiota.

In the absence of intestinal microbiota, IL-10 deficient crypts presented an abnormal amount of metabolically active CBC stem cells, but their PCs reached the end of the cycle. In fact, a greater number of PCs were found in an apparent final state, with a lower number of total granules per cell and a higher percentage of amorphous granules. However, these crypts would not be functional to respond to microbial stimuli. In the presence of gut microbiota, PCs normally increase their secretory activity, their failure rate and therefore, their turnover. Our results suggest that the absence of IL-10 could prevent this process from being carried out successfully. The presence of microorganisms in IL-10^-/-^ mice would lead to a reparative state of the crypt, with an increase in the number of mitotic figures, normalization of the number of CBC stem cells, and an increase in the number of intermediate secretory cells, which do not reach an optimal phenotype of PC.

Finally, it would be possible that the alterations observed in PCs could be a result of a basal inflammatory state of these mice under SPF condition, even though the histological analysis did not show any signs of alterations. It is known the inflammation in IL-10^-/-^ mice is initially driven by a proinflammatory T_H_1 response with an increase of IL-12p40 and IFN-γ [33]. Interestingly, it has been demonstrated that high levels of inflammatory cytokines, such as TNF-α and IFN-γ, can reduce the production of bactericidal peptides [34]. Moreover, Xue and colleagues demonstrated that the reduction of inflammatory markers in IL-10^-/-^ mice by administration of metformin was associated with an improvement in the proliferation and differentiation of the intestinal epithelium [26]. However, we did not detect a higher expression of pro-inflammatory cytokines in IL-10^-/-^ mice compared to WT mice. Only two of six individuals had high levels of IFN-γ in ileum, which suggests that they were beginning to develop intestinal inflammation. These results are highly relevant given that show that alterations in PCs from IL-10^-/-^ mice occur earlier than the symptoms of intestinal inflammation. Probably, PCs alterations could affect the control of the gut microbiota, and at the same time, the resulting colitogenic microbiota together with the inflammatory signals could further alter the state of the crypt.

Additional studies are required to define how IL-10 modulates the phenotype of PCs in mice and to determine whether IL-10 plays equivalent roles in humans. This could help to explain the development of early onset Inflammatory Bowel Disease in patients with IL-10 deficiency.

## Conclusion

Our results show that IL-10^-/-^ mice in SPF conditions have alterations in PCs allocation and maturation, even before developing histological signs of intestinal inflammation. Moreover, the lower expression of bactericidal peptides, together with the impossibility of remodeling the epithelium in response to damage, largely may explain the inflammation developed by IL-10^-/-^ mice after being colonized by microorganisms. Thus, IL-10 could have a key role on the integrity of Lieberkühn crypts, and especially of PCs.

## Supporting information

S1 Table Oligonucleotide sequences used in qRT-PCR analysis. (PDF)

S2 Table Colitis index for IL-10^-/-^ mice exposed to conventional microbiota. (PDF)

S1 Fig Classification of Intermediate cells observed in crypts of Lieberkühn stained with AB-PAS. Yellow arrows show Intermediate Cells (AB+PAS+) classified according to their location, in: adjacent to Paneth cells (P1), in the middle zone of proliferation (P2), or in upper positions over differentiated Goblet Cells (P3).
(PDF)

## References

[1] Fakhoury M, Negrulj R, Mooranian A, Al-Salami H. Inflammatory bowel disease: clinical aspects and treatments. J Inflamm Res. 2014;7: 113–20. 10.2147/JIR.S65979 25075198 PMC4106026

[2] Baumgart DC, Sandborn WJ. Crohn’s disease. Lancet. Elsevier Ltd; 2012;380: 1590–605. 10.1016/S0140-6736(12)60026-9 22914295

[3] De Souza HSP, Fiocchi C. Immunopathogenesis of IBD: current state of the art. Nat Rev Gastroenterol Hepatol. 2016;13: 13–27. 10.1038/nrgastro.2015.186 26627550

[4] Csöngei V, Járomi L, Sáfrány E, Sipeky C, Magyari L, Faragó B, et al Interaction of the major inflammatory bowel disease susceptibility alleles in Crohn’s disease patients. World J Gastroenterol. 2010;16: 176–83. Available: http://www.ncbi.nlm.nih.gov/pubmed/20066736 20066736 10.3748/wjg.v16.i2.176 PMC2806555

[5] Glocker E-O, Kotlarz D, Klein C, Shah N, Grimbacher B. IL-10 and IL-10 receptor defects in humans. Ann N Y Acad Sci. 2011;1246: 102–7. 10.1111/j.1749-6632.2011.06339.x 22236434

[6] Cario E. Innate immune signalling at intestinal mucosal surfaces: a fine line between host protection and destruction. Curr Opin Gastroenterol. 2008;24: 725–32. 10.1097/MOG.0b013e32830c4341 19122523

[7] Pamer EG. Immune responses to commensal and environmental microbes. Nat Immunol. 2007;8: 1173–8. 10.1038/ni1526 17952042

[8] Takaishi H, Matsuki T, Nakazawa A, Takada T, Kado S, Asahara T, et al Imbalance in intestinal microflora constitution could be involved in the pathogenesis of inflammatory bowel disease. Int J Med Microbiol. 2008;298: 463–72. 10.1016/j.ijmm.2007.07.016 17897884

[9] Chassaing B, Darfeuille-Michaud A. The commensal microbiota and enteropathogens in the pathogenesis of inflammatory bowel diseases. Gastroenterology. 2011;140: 1720–28. 10.1053/j.gastro.2011.01.054 21530738

[10] Kühn R, Löhler J, Rennick D, Rajewsky K, Müller W. Interleukin-10-deficient mice develop chronic enterocolitis. Cell. 1993;75: 263–74. 10.1016/0092-8674(93)80068-p 8402911

[11] Sellon RK, Tonkonogy S, Schultz M, Dieleman LA, Grenther W, Balish E, et al Resident enteric bacteria are necessary for development of spontaneous colitis and immune system activation in interleukin-10-deficient mice. Infect Immun. 1998;66: 5224–31. Available: http://www.pubmedcentral.nih.gov/articlerender.fcgi?artid=108652&tool=pmcentrez&rendertype=abstract 9784526 10.1128/iai.66.11.5224-5231.1998 PMC108652

[12] Dieleman LA, Arends A, Tonkonogy SL, Goerres MS, Craft DW, Grenther W, et al Helicobacter hepaticus does not induce or potentiate colitis in interleukin-10-deficient mice. Infect Immun. 2000;68: 5107–13. Available: http://www.ncbi.nlm.nih.gov/pubmed/10948132 10948132 10.1128/iai.68.9.5107-5113.2000 PMC101749

[13] Van der Linde K, Boor PPC, Sandkuijl LA, Meijssen MAC, Savelkoul HFJ, Wilson JHP, et al A Gly15Arg mutation in the interleukin-10 gene reduces secretion of interleukin-10 in Crohn disease. Scand J Gastroenterol. 2003;38: 611–7. Available: http://www.ncbi.nlm.nih.gov/pubmed/12825869 12825869

[14] Zhu H, Lei X, Liu Q, Wang Y. Interleukin-10-1082A/G polymorphism and inflammatory bowel disease susceptibility: A meta-analysis based on 17,585 subjects. Cytokine. 2013;61: 146–153. 10.1016/j.cyto.2012.09.009 23046617

[15] Lin Z, Wang Z, Hegarty JP, Lin TR, Wang Y, Deiling S, et al Genetic association and epistatic interaction of the interleukin-10 signaling pathway in pediatric inflammatory bowel disease. World J Gastroenterol. 2017;23: 4897 10.3748/wjg.v23.i27.4897 28785144 PMC5526760

[16] Vaishnava S, Behrendt CL, Ismail AS, Eckmann L, Hooper LV. Paneth cells directly sense gut commensals and maintain homeostasis at the intestinal host-microbial interface. Proc Natl Acad Sci U S A. 2008;105: 20858–63. 10.1073/pnas.0808723105 19075245 PMC2603261

[17] Salzman NH, Bevins CL. Dysbiosis—a consequence of Paneth cell dysfunction. Semin Immunol. Elsevier Ltd; 2013;25: 334–41. 10.1016/j.smim.2013.09.006 24239045

[18] Adolph TE, Tomczak MF, Niederreiter L, Ko H-J, Böck J, Martinez-Naves E, et al Paneth cells as a site of origin for intestinal inflammation. Nature. 2013;503: 272–6. 10.1038/nature12599 24089213 PMC3862182

[19] Schultz BM, Salazar GA, Paduro CA, Pardo-Roa C, Pizarro DP, Salazar-Echegarai FJ, et al Persistent Salmonella enterica serovar Typhimurium Infection Increases the Susceptibility of Mice to Develop Intestinal Inflammation. Front Immunol. 2018;9: 1166 10.3389/fimmu.2018.01166 29896196 PMC5986922

[20] Cadwell K, Liu JY, Brown SL, Miyoshi H, Loh J, Lennerz JK, et al A key role for autophagy and the autophagy gene Atg16l1 in mouse and human intestinal Paneth cells. Nature. 2008;456: 259–63. 10.1038/nature07416 18849966 PMC2695978

[21] King SL, Mohiuddin JJ, Dekaney CM. Paneth cells expand from newly created and preexisting cells during repair after doxorubicin-induced damage. AJP Gastrointest Liver Physiol. 2013;305: G151–G162. 10.1152/ajpgi.00441.2012 23660502 PMC3725683

[22] Alberts B, Johnson A, Lewis J, Morgan D, Raff M, Roberts K, et al. Molecular Biology of THE CELL. 6th Editio. Garland Science; 2015.

[23] Liu B, Gulati AS, Cantillana V, Henry SC, Schmidt EA, Daniell X, et al Irgm1-deficient mice exhibit Paneth cell abnormalities and increased susceptibility to acute intestinal inflammation. Am J Physiol Gastrointest Liver Physiol. 2013;305: G573–84. 10.1152/ajpgi.00071.2013 23989005 PMC3798734

[24] Varnat F, Heggeler BB-T, Grisel P, Boucard N, Corthésy-Theulaz I, Wahli W, et al PPARbeta/delta regulates paneth cell differentiation via controlling the hedgehog signaling pathway. Gastroenterology. 2006;131: 538–53. 16890607 10.1053/j.gastro.2006.05.004

[25] Natividad JMM, Hayes CL, Motta J-P, Jury J, Galipeau HJ, Philip V, et al Differential induction of antimicrobial REGIII by the intestinal microbiota and Bifidobacterium breve NCC2950. Appl Environ Microbiol. 2013;79: 7745–54. 10.1128/AEM.02470-13 24096422 PMC3837813

[26] Xue Y, Zhang H, Sun X, Zhu M-J. Metformin Improves Ileal Epithelial Barrier Function in Interleukin-10 Deficient Mice. Han X, editor. PLoS One. 2016;11: e0168670 10.1371/journal.pone.0168670 28002460 PMC5176295

[27] Kosinski C, Stange DE, Xu C, Chan AS, Ho C, Yuen ST, et al Indian hedgehog regulates intestinal stem cell fate through epithelial-mesenchymal interactions during development. Gastroenterology. NIH Public Access; 2010;139: 893–903. 10.1053/j.gastro.2010.06.014 20542037 PMC2930094

[28] He XC, Zhang J, Tong W-G, Tawfik O, Ross J, Scoville DH, et al BMP signaling inhibits intestinal stem cell self-renewal through suppression of Wnt–β-catenin signaling. Nat Genet. 2004;36: 1117–1121. 10.1038/ng1430 15378062

[29] Demidova-Rice TN, Hamblin MR, Herman IM. Acute and impaired wound healing: pathophysiology and current methods for drug delivery, part 1: normal and chronic wounds: biology, causes, and approaches to care. Adv Skin Wound Care. NIH Public Access; 2012;25: 304–14. 10.1097/01.ASW.0000416006.55218.d0 22713781 PMC3428147

[30] Quiros M, Nishio H, Neumann PA, Siuda D, Brazil JC, Azcutia V, et al Macrophage-derived IL-10 mediates mucosal repair by epithelial WISP-1 signaling. J Clin Invest. 2017;127: 3510–3520. 10.1172/JCI90229 28783045 PMC5669557

[31] Hasnain SZ, Tauro S, Das I, Tong H, Chen AC-H, Jeffery PL, et al IL-10 promotes production of intestinal mucus by suppressing protein misfolding and endoplasmic reticulum stress in goblet cells. Gastroenterology. 2013;144: 357–368.e9. 10.1053/j.gastro.2012.10.043 23123183

[32] Kishore R, Krishnamurthy P, Garikipati VNS, Benedict C, Nickoloff E, Khan M, et al Interleukin-10 inhibits chronic angiotensin II-induced pathological autophagy. J Mol Cell Cardiol. 2015;89: 203–13. 10.1016/j.yjmcc.2015.11.004 26549357 PMC4689660

[33] Berg DJ, Davidson N, Kühn R, Müller W, Menon S, Holland G, et al Enterocolitis and colon cancer in interleukin-10-deficient mice are associated with aberrant cytokine production and CD4(+) TH1-like responses. J Clin Invest. American Society for Clinical Investigation; 1996;98: 1010–20. 10.1172/JCI118861 8770874 PMC507517

[34] Schaubeck M, Clavel T, Calasan J, Lagkouvardos I, Haange SB, Jehmlich N, et al Dysbiotic gut microbiota causes transmissible Crohn’s disease-like ileitis independent of failure in antimicrobial defence. Gut. 2016;65: 225–237. 10.1136/gutjnl-2015-309333 25887379 PMC4752651

