# Supplementary material for: The absence of interleukin 10 affects the morphology, differentiation, granule content and the production of cryptidin-4 in Paneth cells in mice
Source: PLoS One. 2019 Sep 11;14(9):e0221618. doi: 10.1371/journal.pone.0221618 (PMC6738610; doi:10.1371/journal.pone.0221618)
Supplement: S1 Table — (PDF) [file pone.0221618.s001.pdf]

	Sense	Antisense
Cryptdin-1	CTA GTC CTA CTC TTT GCC CT	TTG CAG CCT CTT GAT CTA CA
Cryptdin-4	GTC CAG GCT GAT CCT ATC CA	GGG GCA GCA GTA CAA AAA TC
RegIIIγ	TTC CTG TCC TCC ATG ATC AAA A	CAT CCA CCT CTG TTG GGT TCA
Lysozyme	CAA GAT CTA AGA ATG CCT GTG	TTC CGA ATA TAC TGG GAC AG
GAPDH	TGA AGC AGG CAT CTG AGG G	CGA AGG TGG AAG AGT GGG AG
