# Supplementary material for: The absence of interleukin 10 affects the morphology, differentiation, granule content and the production of cryptidin-4 in Paneth cells in mice
Source: PLoS One. 2019 Sep 11;14(9):e0221618. doi: 10.1371/journal.pone.0221618 (PMC6738610; doi:10.1371/journal.pone.0221618)
Supplement: S2 Table — (PDF) [file pone.0221618.s002.pdf]

Observations	Score
Grade 0: Normal appearance of anus and feces.	0
Grade 1: Mild inflammation of anus, perirectal edema, occasional tenesmus and feces with normal or soft consistency.	1
Grade 2: Interment rectal prolapse, tenesmus, rectal edema, tenesmus feces with normal or soft consistency, mice need to be monitored more.	2
Grade 3: Permanent prolapse, congestion and edema on rectal mucosa, diarrhea or absence of feces, blood on feces. Immediate euthanasia is advised.	3
