# Supplementary figures and images for: The absence of interleukin 10 affects the morphology, differentiation, granule content and the production of cryptidin-4 in Paneth cells in mice
Source: PLoS One. 2019 Sep 11;14(9):e0221618. doi: 10.1371/journal.pone.0221618 (PMC6738610; doi:10.1371/journal.pone.0221618)

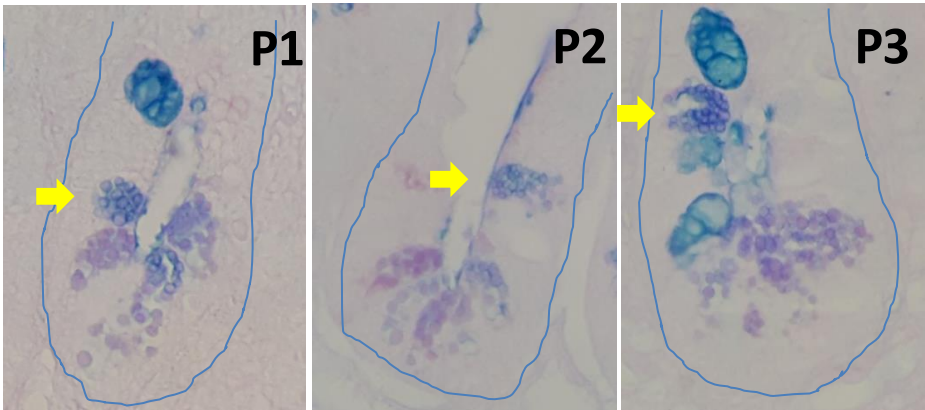

Supplement: S1 Fig — Yellow arrows show Intermediate Cells (AB+PAS+) classified according to their location, in: adjacent to Paneth cells (P1), in the middle zone of proliferation (P2), or in upper positions over differentiated Goblet Cells (P3). (PDF) [file pone.0221618.s003.pdf]
